# Supplementary material for: Heat adaptation of phage T7 under an extended genetic code
Source: Virus Evol. 2021 Dec 1;7(2):veab100. doi: 10.1093/ve/veab100 (PMC8923235; doi:10.1093/ve/veab100)
Supplement: veab100_Supp [file veab100_supp.zip › Sequencing_Analysis.zip!/Sequencing_Analysis/S2/marginal.html]

BRESEQ :: Marginal Predictions


|  |  |
| --- | --- |
|  | ***breseq***  version 0.30.1    mutation predictions \| marginal predictions \| summary statistics \| genome diff \| command line log |

| Marginal read alignment evidence | | | | | | | | | | | |
| --- | --- | --- | --- | --- | --- | --- | --- | --- | --- | --- | --- |
|  | seq id | position | | ref | new | freq | score (cons/poly) | reads | annotation | genes | product |
| \* | NC\_001604.1 | 37,346 | 0 | T | C | 61.3% | 4000.4 / inf | 5152 | intergenic (–/–) | *–/–* | –/– |
| \* | NC\_001604.1 | 35,356 | 0 | T | G | 54.0% | 3129.3 / inf | 5417 | intergenic (–/–) | *–/–* | –/– |
| \* | NC\_001604.1 | 35,515 | 0 | A | C | 48.1% | 1287.0 / inf | 5686 | intergenic (–/–) | *–/–* | –/– |
| \* | NC\_001604.1 | 35,254 | 0 | A | G | 37.3% | 3426.1 / inf | 4876 | intergenic (–/–) | *–/–* | –/– |
| \* | NC\_001604.1 | 33,279 | 0 | T | C | 33.1% | 4707.9 / inf | 4989 | intergenic (–/–) | *–/–* | –/– |
| \* | NC\_001604.1 | 36,918 | 0 | T | C | 31.2% | 5428.1 / inf | 5123 | intergenic (–/–) | *–/–* | –/– |
| \* | NC\_001604.1 | 24,238 | 0 | A | G | 28.8% | 6279.6 / inf | 5215 | intergenic (–/–) | *–/–* | –/– |
| \* | NC\_001604.1 | 35,366 | 0 | C | A | 27.9% | 8309.4 / inf | 5408 | intergenic (–/–) | *–/–* | –/– |
| \* | NC\_001604.1 | 160 | 0 | T | G | 26.5% | 4673.1 / inf | 4467 | intergenic (–/–) | *–/–* | –/– |
| \* | NC\_001604.1 | 33,023 | 0 | A | G | 25.0% | 9424.4 / inf | 6553 | intergenic (–/–) | *–/–* | –/– |
| \* | NC\_001604.1 | 33,069 | 0 | G | T | 23.0% | 13164.9 / inf | 6771 | intergenic (–/–) | *–/–* | –/– |

| Marginal new junction evidence (lowest skew 10 of 39 shown) | | | | | | | | | | | |
| --- | --- | --- | --- | --- | --- | --- | --- | --- | --- | --- | --- |
|  | | seq id | position | reads (cov) | reads (cov) | score | skew | freq | annotation | gene | product |
| \* | ? | NC\_001604.1 | = 36882 | 5203 (0.980) | 883 (0.170) | 243/582 | 5.8 | 15.5% | intergenic (–/–) | *–/–* | –/– |
|  | ? | NC\_001604.1 | 37265 = | 4556 (0.890) |  |  |  |  | intergenic (–/–) | *–/–* | –/– |
| \* | ? | NC\_001604.1 | = 19585 | 4495 (0.850) | 324 (0.060) | 168/586 | 12.2 | 6.9% | intergenic (–/–) | *–/–* | –/– |
|  | ? | NC\_001604.1 | 19598 = | 4417 (0.850) |  |  |  |  | intergenic (–/–) | *–/–* | –/– |
| \* | ? | NC\_001604.1 | = 16508 | 3993 (0.750) | 107 (0.020) | 85/586 | 24.4 | 2.8% | intergenic (–/–) | *–/–* | –/– |
|  | ? | NC\_001604.1 | 16970 = | 3426 (0.660) |  |  |  |  | intergenic (–/–) | *–/–* | –/– |
| \* | ? | NC\_001604.1 | = 19635 | 4894 (0.920) | 35 (0.010) | 29/590 | 41.2 | 0.72% | intergenic (–/–) | *–/–* | –/– |
|  | ? | NC\_001604.1 | 19645 = | 4786 (0.920) |  |  |  |  | intergenic (–/–) | *–/–* | –/– |
| \* | ? | NC\_001604.1 | = 281 | 5233 (1.110) | 1199 (0.240) +TAAAGACCAGACC | 21/508 | 41.8 | 17.7% | intergenic (–/–) | *–/–* | –/– |
|  | ? | NC\_001604.1 | 282 = | 5794 (1.090) |  |  |  |  | intergenic (–/–) | *–/–* | –/– |
| \* | ? | NC\_001604.1 | = 8335 | 5558 (1.050) | 25 (0.000) | 22/584 | 44.4 | 0.45% | intergenic (–/–) | *–/–* | –/– |
|  | ? | NC\_001604.1 | 8939 = | 5643 (1.100) |  |  |  |  | intergenic (–/–) | *–/–* | –/– |
| \* | ? | NC\_001604.1 | = 13586 | 0 (0.000) | 46 (0.010) | 20/592 | 45.7 | 50.3% | intergenic (–/–) | *–/–* | –/– |
|  | ? | NC\_001604.1 | 14356 = | 91 (0.020) |  |  |  |  | intergenic (–/–) | *–/–* | –/– |
| \* | ? | NC\_001604.1 | = 13568 | 61 (0.010) | 36 (0.010) | 16/588 | 48.0 | 43.4% | intergenic (–/–) | *–/–* | –/– |
|  | ? | NC\_001604.1 | 14352 = | 34 (0.010) |  |  |  |  | intergenic (–/–) | *–/–* | –/– |
| \* | ? | NC\_001604.1 | 295 = | 7072 (1.340) | 31 (0.010) | 13/560 | 48.9 | 0.47% | intergenic (–/–) | *–/–* | –/– |
|  | ? | NC\_001604.1 | = 281 | 6648 (1.350) |  |  |  |  | intergenic (–/–) | *–/–* | –/– |
| \* | ? | NC\_001604.1 | = 13568 | 61 (0.010) | 33 (0.010) | 13/586 | 49.9 | 51.7% | intergenic (–/–) | *–/–* | –/– |
|  | ? | NC\_001604.1 | 14339 = | 2 (0.000) |  |  |  |  | intergenic (–/–) | *–/–* | –/– |
